# Applying Contemporary Management Principles to Implementing and Evaluating Value-Added Pharmacist Services

**DOI:** 10.3390/pharmacy7030099

**Published:** 2019-07-20

**Authors:** Shane P. Desselle, Leticia R. Moczygemba, Antoinette B. Coe, Karl Hess, David P. Zgarrick

**Affiliations:** 1 California College of Pharmacy, Touro University, Vallejo, CA 94952, USA; 2 College of Pharmacy, University of Texas at Austin, Austin, TX 78712, USA; 3 College of Pharmacy, University of Michigan, Ann Arbor, MI 48109, USA; 4 Keck Graduate Institute, School of Pharmacy and Health Sciences, Claremont, CA 91711, USA; 5 Bouve College of Health Sciences, School of Pharmacy, Northeastern University, Boston, MA 02115, USA

**Keywords:** pharmacist, services marketing, management, value-added services

## Abstract

Value-added pharmacy services encompass traditional and emerging services provided by pharmacists to individual and entire populations of persons increasingly under the auspices of a public health mandate. The success of value-added pharmacy services is enhanced when they are carried out and assessed using appropriate theory-based paradigms. Many of the more important management theories for pharmacy services consider the “servicescape” of these services recognizing the uniqueness of each patient and service encounter that vary based upon health needs and myriad other factors. In addition, implementation science principles help ensure the financial viability and sustainability of these services. This commentary reviews some of the foundational management theories and provides a number of examples of these theories that have been applied successfully resulting in a greater prevalence and scope of value-added services being offered.

## 1. Introduction

Pharmacists have always been in a service industry, including those in the community sector selling medicines and other health products, specifically for the delivery of health and medication-related services. The role of the pharmacist has evolved over the years, from compounding remedies from raw materials; to dispensing pre-manufactured dosage forms; to educating, advising, managing, and monitoring the outcomes of drug therapy for both patients and populations. All of these services add value to medication and patient care outcomes. Additionally, pharmacists have continued to develop services that add value to the medication use process and encompass a variety of individual and public health services. The more “traditional” and newly emerging services can be regarded as “value-added” services, or those that are likely to positively impact medication outcomes [1]. These services are especially salient for certain populations of vulnerable or high-risk persons, such as those with multiple comorbidities, younger and older patients, those with diminished access to care, those with marginal or low health literacy, and the medically underserved, to name a few. As such, value-added services aim to contribute toward and improve public health and health outcomes across diverse settings. Among the more common value-added services of pharmacists and pharmacy personnel under the aegis of public health is the provision of immunizations for various infectious diseases, such as for influenza, herpes zoster and pneumococcal [2] as well as provision of education and advocacy related to vaccinations [3,4]. Pharmacists are also increasing their roles in public health issues such as health literacy [5,6] opioid misuse and naloxone training and education [7,8,9,10], emergency preparedness planning [11] and safe medication disposal [12,13]. Pharmacists are also members of health care teams in ambulatory care settings and provide medication and disease management for chronic conditions such as diabetes, hypertension, and congestive heart failure [14,15,16,17]. In the hospital setting, pharmacists have roles in antibiotic stewardship [18,19], medication reconciliation [20,21] and therapeutic drug monitoring [22,23].

## 2. Business Planning for Successful Implementation of Pharmacist Services

Careful planning and management is needed to evaluate whether a value-added pharmacist service should be pursued or not. Thus, the creation of a business plan is a foundational step in beginning a new service. A business plan helps stakeholders (e.g., administrators, investors, payers, other health care professionals) decide whether to devote resources and energy to a new service. The first step of a business plan is to identify how a value-added service aligns with an organization’s strategic plan, mission statement, and current priority areas. For example, an organization that is focused on improving diabetes outcomes will likely not be interested in a service that focuses on asthma [24]. Next, input from stakeholders such as pharmacists, technicians, physicians and other health professionals, and patients should be sought to refine ideas and tailor a new service to stakeholders’ needs. Given that pharmacists have an established track record of implementing services ranging from medication therapy management to chronic disease management to immunization programs, it is likely that a review of the primary literature and pharmacy organization websites would provide guidance and examples of successful models that could be replicated [24].

It is also worthwhile to consider the flexibility level of the pharmacy organization when deciding what type of value-added service to pursue. Feletto has described four states of flexibility that a pharmacy organization may be in when transitioning to provision of value-based services. These include steady-state, operational, structural, and strategic flexibility [25]. A pharmacy in steady-state does not offer services beyond traditional dispensing activities. Operational flexibility occurs when a pharmacy is beginning to expand offerings to increase the number of customers but not providing a value-based service. A pharmacy that has developed at least one value-based service and begun to implement internal infrastructure to support the service is exhibiting structural flexibility, although at this point there is not a plan for long-term success. Strategic flexibility occurs when a pharmacy has successfully implemented value-based services and has infrastructure and processes in place for sustainability [25]. A pharmacy in a steady-state may venture into value-based services by offering immunization services whereas a pharmacy in strategic flexibility may be in a position to partner with providers for a diabetes management service.

## 3. The Market for Pharmacist Services

No matter how valuable the service might be in the mind of the pharmacist and no matter how well executed, if that service does not fulfill a perceived or actual need by its target market, it will likely fail. Thus, it is essential to consider the target market for the service. Collaboration with other health care professionals is usually needed for a successful pharmacist service [24,26]. Building relationships with health care professionals who could benefit from a pharmacist service is important to facilitate patient referrals, medical information exchange, and billing for pharmacist services. Pharmacists and health care providers are likely aware of each other in a community but that does not automatically translate to meaningful collaboration. Thus, pharmacists will need to spend some effort increasing interactions with local providers to develop successful services. Once a provider recognizes that a pharmacist can provide nondispensing services, a pharmacist can begin to build trust and rapport to lead to sharing of patient information, referrals, etc. To build rapport it is important to be able to clearly articulate the purpose of the service and how it benefits a health care professional, such as a physician. For example, a pharmacist seeking referrals for medication therapy management in a community setting may highlight benefits such as improved medication adherence which can in turn lead to a decrease in use of acute services such as hospitalization. Physicians are under increasing pressure to meet quality metrics such as controlling high blood pressure in their patients, and this creates opportunities to align value-based pharmacist services with blood pressure monitoring and medication therapy management [24].

Payment for services should always be considered when thinking about a new pharmacist service. Thus, health care payers are another stakeholder to consider when marketing a new service. Pharmacists have had some success with payers reimbursing for nondispensing services such as medication therapy management (MTM) and diabetes management [27,28]. However, the fact that pharmacists are not recognized as providers by most payers can present challenges to pharmacists negotiating service payment. It is important that pharmacist services align with priorities of payers, which often means reducing high-cost expenditures such as emergency department visits or hospitalizations for chronic conditions. The “value-add” of a service for a payer often means decreasing costs while improving patient outcomes [29,30]. Effective management would entail understanding of various reimbursement mechanisms and leveraging them not only for the success of the pharmacy, but perhaps even a larger organization. For example, Wu et al. used monies acquired for participating in the 340B drug discount program to create a financially viable and more clinically relevant hospital discharge service associated with improved patient outcomes, such as a reduction in 30-day readmissions [31].

A market evaluation should also consider the competition for services. Depending upon the type of service being provided, competitors could include physicians, nurse practitioners, and even other pharmacists/pharmacies [24,32]. In addition to pharmacists, diabetes education can be provided by physicians, nurse practitioners, and other health professionals. A payer would want to know why a pharmacist versus other qualified health professional should provide diabetes education to its beneficiaries. In a community pharmacy, perhaps the value-add is the convenience of providing the service on a walk-in basis with little to no waiting whereas in a patient-centered medical home, the value-add could be increasing physician time to focus on other patients or services. To effectively mitigate the impact of competitors, a pharmacist should be able to describe how a service is unique from similar services [24,33].

The viability of a pharmacist service is also impacted by the volume of potential consumers (i.e., patients) in its service area. Internal and external data can be used to help assess market size for a pharmacist service. Internal data, such as patient profiles, purchasing, and financial records can help examine the number of potentially eligible patients within the organization. Often, a new service has a goal of attracting new patients. In this case, local information about desired health outcomes can be obtained from interviews of other health care professionals in the pharmacy’s market area or a review of the local health department website to identify indications and prevalence of the area’s major health burdens. A market research study could also be undertaken to collect feedback and perceptions about a service from potential patients [24,34,35].

Although market research may indicate patient interest in a particular service, ultimately patients may not be willing to pay for a service if they cannot afford it or if they do not perceive value in the service [24,29]. This may be especially true if a service is not covered by insurance and a patient would have to pay out-of-pocket. Although Medicare recognizes pharmacists as a provider for MTM provided to Medicare Part D beneficiaries and some state Medicaid programs and commercial payers will pay for pharmacist services, most payers do not pay for pharmacist services in the United States [29]. Government sponsorship helps cover costs for other services in other countries such as U.K.’s minor ailment service [36] and Australia’s Home Medicines Review program [37].

## 4. Patient Engagement and Participation in Services Delivery

Consumers’ willingness to pay and even their impressions and experiences with a service will likely be enhanced if they are engaged and participatory in the service encounter [38]. For example, if a pharmacist has effectively engaged a patient in a lipid management service, the patient will likely be more successful in the endeavor to maintain control, or keep their lipid levels below target goal. Further, if the pharmacist is able to get that same patient to relay successes and strategies for how they are succeeding and engage them in provocative discussion, then the patient will enjoy the entirety of the service to a greater degree. This behooves the pharmacist to ascertain the degree that patients might want to engage, thus tailoring strategies of service delivery to reflect patient desires. As such, any service designed must be flexible, and pharmacists and support personnel providing the service must be adaptive to the situation. With this in mind, research has demonstrated that the transtheoretical model (TTM), health belief model, and theory of planned behavior can be useful guides for pharmacists in designing and implementing value-added services [39]. This extends toward contemporary patient needs and public issues, such as that with the opioid crisis. Fleming et al. effectively employed the theory of planned behavior to identify challenges to overcome in pharmacists’ engagement of patients in an attempt to curb their misuse of drug substances [40].

## 5. Meeting Patient Needs

Assuming that there is a consumer need and a market for a particular value-added pharmacist service, business planners must ascertain whether the service is meeting a need. One way to discern this is through a SWOT (i.e., strengths, weaknesses, opportunities, and threats) analysis [24]. In a SWOT analysis, internal opportunities and weaknesses and external opportunities and threats are examined. Generally, internal opportunities and weaknesses are easier to control than external opportunities and threats. Internally, resources such as personnel, training requirements for pharmacists and staff, space, equipment (e.g., computers and software) and supplies (e.g., medical and office supplies) needed to deliver a service should be determined. This can help decide the amount of working capital necessary to support development of a new service. Initially, an organization may leverage existing resources, such as reallocation of pharmacist time to include a percentage of time to deliver MTM, before investing large amounts of capital into a service. At a minimum, resources for equipment, supplies, and market are often needed [24]. Administrative support and buy-in for a new service is critical to getting support for resources needed for the service. Evaluating the culture is also part of the homework that needs to be done. Organizational culture has shown to be the primary driver in the success of many value-added pharmacy services. The organization’s culture might or might not be receptive to new ideas, might infer a preference as to who or what types of persons approach upper administration with ideas, and also how to couch, or frame the discussion when pitching the idea [41].

Externally, a number of factors, such as demographic trends of patients and providers, state scope of practice laws and regulations, and the number and type of competitors in a market, can represent opportunities or threats for a value-added pharmacist service. For example, an aging population might represent an opportunity for disease management, but a threat in lack of time to devote to non-dispensing activities. If a pharmacist has identified the potential opportunities or threats to a service, they can apply some of the management principles described above to manage the impact.

Patients often understand their own needs, but in some cases, might not perceive a need for a particular service or even see its relevance unless they have been properly engaged. While some patients are undertreated, many patients with comorbid disease states are on too many medications that may result in untoward outcomes, even idiosyncratic conditions. Historically referred to as “polypharmacy”, the term “deprescribing” refers to systematic and evidence-based practices to deprescribe, or streamline a patient’s therapeutic regiment to maximize outcomes. Trenament et al. described a service in which they engaged patients in a deprescribing program to promote those patients’ buy-in, have greater confidence in and thus better adherence to their new medication regimen [42].

## 6. Managing the Servicescape

The ability of pharmacists to manage internal and even some external factors in service deliveries provides them great opportunities in what has been referred to as the “servicescape” [43]. It refers to physical and other constructs wherein a service is performed, delivered, and consumed [44]. There are objective stimuli generated during the performance and consumption/use of a service that are measurable and controllable to enhance employee/patient interactions. The servicescape influences the patient’s experience and thus their satisfaction with service experience. For example, the layout and structural design of the pharmacy, along with job descriptions and workflow, will determine where a patient drops off their prescription, how long it is they might have to wait before being greeted, who takes responsibility for greeting them and for initial medication history-taking and other services, and the extent to which privacy can be offered and even the types or array of services that could potentially be offered for each patient. In the background, various “physical” attributes ranging from such phenomena even as the type [or lack] of music playing in the background of the pharmacy has an impact on the overall sensory experience of the patient. Design of the servicescape can be informed by any number of questions such as:

How can the servicescape be designed to attract the most profitable customer/patient segments to the service? 

How can the servicescape be designed to maximize customer satisfaction and retention? 

How much money and resources should be invested into the servicescape? These questions get to the core business mission, vision, and values of the organization and thus translate into consideration of which service, or services might be offered from an array of possibilities.

## 7. Array of Value-Added Pharmacist Services

Value-added pharmacist services range from focused, one-time interventions to resolve medication issues identified during the dispensing process to nondispensing direct patient care services that focus on comprehensive and longitudinal management of medications for chronic conditions. Prevention and wellness services are another type of pharmacist service [24].

Recently, there has been a focus on creating opportunities in the community pharmacy setting for increased engagement between pharmacists and patients and to enhance clinical services [44]. Continuous medication monitoring (CoMM) is one approach to that has been gaining momentum [45]. In CoMM, “pharmacists systematically review the patients' medication record and monitor every medication being dispensed to prevent, identify, and resolve drug therapy problems or obstacles to optimal therapy during the dispensing process” [45]. Traditional MTM, especially for Medicare Part D beneficiaries whereby pharmacists can be reimbursed, remains a clinical service offered in many pharmacies. MTM generally includes five key steps, including comprehensive medication review, medication action plan, personalized medication record, intervention and referral, and documentation and follow-up [46].

Value-added services, such as appointment-based models (ABM), may be integrated with existing services such as CoMM or MTM. The goal of ABM is to increase medication adherence and efficiency for pharmacies and patients [47]. ABM is comprised of three components: medication synchronization which includes refilling all medications on the same day each month, monthly phone call to patient to confirm refill order and identify any medication-related issues, and scheduled monthly appointment to pick up the medications [48]. At the monthly appointment, additional services such as MTM may be provided to address medication-related problems.

Whereas MTM is a holistic approach to managing all medications, disease state management is a more focused type of service that pharmacists provide. In disease management, pharmacists educate and monitor medication therapy to achieve therapy goals over a period of time, make recommendations about drug therapy to providers, and may be able to directly initiate or change therapy under a collaborative practice agreement (depending upon each state’s scope of practice). Examples of conditions that pharmacists commonly manage include hypertension [49], heart failure [50], diabetes [51,52], and asthma [53,54].

Finally, pharmacists have an established history of delivering monitoring/screening services and wellness/health-promotion programs. Examples include anticoagulation services [54,55,56,57], travel health [55,58], hormonal contraception prescribing [59], naloxone dispensing and education [60], smoking cessation counseling [61], osteoporosis screenings [62], and lipid screenings [63]. Point-of-care testing for conditions such as influenza, strep throat, and hepatitis C is also increasing [64,65].

## 8. The Aesthetics of Services Delivery and Consumption

There are various idioms referring to the importance of making a good first impression, or a good impression, overall. Service companies depend on front-line service workers to control and communicate a certain image that consumers/patients will associate with the business and with the service [66]. In community practice, technicians have been referred to by pharmacists as the “face” of the pharmacy [67]. Image control demands that service workers act according to scripts that diverge from their actual preferences and capacities. Additionally, service recipients are also acting on social norms, as well as to gauge the intent of the service provider and to potentially acquire a higher level of service or enhance their own experiences. Given the acting of both parties, a number of conflicts arise regarding the truthfulness of sincerity regarding service performance. The expected set of behaviors each actor in the process plays out are often referred to as roles; and thus role theory might be helpful in determining proper training for pharmacy staff and for the expectations one might have of patients. The aesthetic appeal of services is thus under significant control and can be managed effectively by the pharmacy. This includes ascribing proper roles to workers, training patients on the roles they might assume in receiving services, and strategizing to optimize the impression by patients of the service. The impression of those using or experiencing the service will go a long way toward successful marketing of the service, particularly in an age where reviews of experiences are proliferated so quickly on web-based ratings platforms. It is important, then, to manage not only the entirety or “gestalt” of the service but also its individual components. A recent study of both “traditional” and emerging pharmacist services found that visual appeal of the pharmacy and its aesthetics, including the image projected by service personnel (pharmacists and technicians) impacts the relationship between perceived quality of the service and thus customer loyalty [68].

## 9. Components of A Value-Added Pharmacist Service

Once the decision is made to implement a new service, patient eligibility criteria for the service needs to be established and roles and responsibilities of the pharmacist and other supporting staff need to be defined and agreed upon by all stakeholders [69,70]. Patient eligibility will vary depending upon the service but may include patients who are not meeting goals for a particular chronic disease, patients with multiple chronic conditions, or patients taking multiple chronic medications [66]. Also, workflow for delivery of the intervention and processes such as identification of eligible patients, data collection, and documentation, needs to be developed. A detailed workflow can help guide next steps needed for implementation such as reallocation of existing pharmacist resources to deliver the service or engaging information technology personnel to create a flag in an electronic health record to identify eligible patients [71].

### 9.1. Patient Data Collection

A well-developed data collection plan is important to ensure that relevant baseline and monitoring variables are systematically recorded when the service is provided. A pharmacy management system or electronic health record may need to be modified in order to collect the desired data [24]. This process should begin early because it can be time-intensive as it often involves additional organizational approvals and may depend upon availability of personnel from nonpharmacy departments such as information technology [72]. A data collection plan should also include details about security of data and compliance with rules and regulations such as the Health Insurance Portability and Accountability Act of 1996 HIPAA [24].

### 9.2. Pharmacy-Based Laboratory 

While laboratory data are often obtained from external sources, it may be advantageous or even necessary to conduct laboratory monitoring/screening as part of a broader service. Thus, some pharmacists have equipment and supplies available to measure blood glucose, A1C, international normalization ratio, lipids, or bone mineral density at the point-of-care. Any pharmacy performing tests that involve collecting blood or saliva must follow guidelines according to the Clinical Laboratory Improvement Amendments of 1988. Other considerations include being familiar with the Occupational Safety and Health Act (OSHA) and having a plan in place for blood-borne pathogen exposure [24]. In some U.S. states, pharmacists are permitted to order laboratory tests when needed to assess and monitor medication-related problems [73].

### 9.3. Medication Management Protocols/Collaborative Practice Agreements

A protocol or collaborative practice agreement (CPA) is useful to provide a framework for consistent delivery and guide treatment decisions for a medication or disease management service, which typically includes a comprehensive assessment of medications for appropriateness and whether or not treatment goals are being reached, identification and resolution of medication-related problems, development of a medication care plan and follow-up and monitoring [74,75]. Protocols or CPAs formalize collaborations between pharmacists and providers and “define certain patient care functions that a pharmacist can autonomously provide under specified situations and conditions” [76].Treatment pathways in protocols or CPAs should adhere to national guidelines and evidence-based recommendations and reflect input and feedback from collaborating providers, such as physicians and nurse practitioners. The roles and responsibilities of each team member should also be clearly defined. While a protocol or CPA is typically not required for pharmacist services, it is advantageous to outline a process to support decision-making. Further, a protocol or CPA improves efficiency of a pharmacist being able to directly implement therapy recommendations rather than wait for provider approval [24].

CPAs, or as they are otherwise known as collaborative practice agreements (CPAs), can become so commonplace and so successful as to eventually actuate or serve as a foundation for changes in scope of practice. For example, Farris et al. utilized a CPA to extend pharmacists’ roles in providing oral contraceptive medications and other related services [77]. This and other programs like it, along with regulatory and societal attitude evolutions about oral contraception, have resulted in more commonplace pharmacist involvement in such services [78].

### 9.4. Patient Education

Patient education is commonly included as part of a value-added pharmacist service although the extent of education varies by the type of service being delivered. Patient education may be brief such as discussing benefits/risks of immunizations for an immunization service or providing one-time education about how a medication works and expected outcomes from taking a medication [24]. Pharmacists are increasingly expanding their roles in managing patient education services, such as is the case with nutrition education [79] and in engaging patients in successful weight loss programs using individualized and group education, as well as other tailored interventions [80]. More comprehensive education may be provided over time for disease management services. For example, in diabetes management pharmacists may spend time training a patient on how to use a blood glucose monitor and/or insulin injections and providing education about healthy eating, signs and symptoms of hypoglycemia, and assessing patient goals for treatment over multiple visits. For education services, it is important for a pharmacist to consider literacy levels and needs of local populations, such as availability of educational materials in English and Spanish, when applicable [24].

## 10. Outcome Measurements

Pharmacists need to think about which outcomes should be assessed to determine the effectiveness and value of services. It is a good practice to obtain feedback from key stakeholders about outcomes as well. For example, pharmacists often focus on clinical outcomes whereas an administrator may be primarily focused on economic outcomes. A combination of process, clinical, and economic outcomes are useful to be able to have a full picture of the impact of a service. Process measures may include the number and type of medication-related problems identified and resolved. Examples of clinic measures include systolic and diastolic blood pressure, A1C, and lipid levels. Economic outcomes can range from total health care costs to costs of medications, hospitalizations or emergency department visits. Some stakeholders may also be interested in the return-on-investment for a particular service [24]. Pharmacists should determine if desired outcome data is easily available in a pharmacy management system or electronic health record. Often, a pharmacist has to work with information technology personnel to develop templates to systematically collect and document outcomes for reporting purposes [72,81]. Humanistic outcomes, such as patient satisfaction, often require collecting data from patients with a tool such as a survey. For some outcomes, such as cost data, partnering with payers for claims data may be necessary. Thus, it is important to create an evaluation plan early to have the necessary infrastructure in place for successful outcome reporting.

Outcomes are not necessarily mutually exclusive to one another. More often than not, when exemplary clinical and humanistic outcomes are being achieved, financial outcomes are in lock-step with them. As mentioned previously, pharmacies develop services in agreement with their mission and to advance the profession and to diversify their revenue streams. Relying solely on product distribution or even hanging one’s hat on one particular service can be especially problematic in an environment of shrinking profit margins. It is healthy and wise to consider concomitant services that leverage one another in use of resources and in marketing, while also focusing on a comprehensive set of operations management strategies that seek to optimize concurrent and various types of outcomes. It has been suggested that pharmacies develop and succeed in distinctive competencies, ranging from unique services delivery, to distribution efficiency, and marketing efforts to maximize the likelihood of clinical and economic success [82].

## 11. Pharmacist and Staff Training

For a service to be successful and attractive to payers, the service needs to be consistently and systematically delivered to all patients [83]. To ensure this, pharmacists and staff should be trained and proficient in delivering the service. The type and extent of training varies according to the type of service being provided. For services such as MTM, pharmacists may adopt national programs such as the American Pharmacists Association MTM Certificate program [84]. A component of training should also focus on documentation and outcome reporting procedures. It also useful to provide scripts or workflow diagrams (may be electronic or on paper) as part of the training to guide pharmacists in consistent delivery of an intervention. Adequate training of support personnel is demonstrated repeatedly for successful expansion of pharmacist services, such as when technicians accept medication histories of patients being admitted into the hospital, thus affording pharmacists the ability to effectively work with prescribers on medication care planning [85]. Annual booster trainings should also be considered to maintain quality [83]. Technicians and support personnel involved in record-keeping, coordination, and assistance with data retrieval as a component of the service process should receive training as well as the overall goals of the service, how the service fits with the organization’s mission, and what value they (as support staff) bring to the table in executing the service [24].

## 12. Management and Marketing Services—The Services as Theatre Paradigm

There are many ways one can conceptualize services so as to proffer management and marketing best practices for their development and implementation. Grove et al. envisage service as a human drama depicting the service experience [77]. Those performing the service are actors aiming to create a desired impression before the audience on the “front stage” [86]. The “rehearsal” for the performance takes place “backstage” away from the audience and where strategies for design and implementation are laid out. In drama, the service is carried out not in the absence, but rather, with the audience’s input. Similarly, a performance of a service often requires stakeholder (i.e., patients, physicians and other health professionals, and payers) participation in the process. Like theatrical performances, services delivery can be tenuous and fragile as processes can be disrupted by minor mishaps. As such, both service provision and drama performance employ strategies to create a desirable impression. This includes avoidance of service mistakes, of which things like poor service design are obvious (e.g., not aligning a service with organizational needs), but also avoidance of “simple” errors like misspelling a patient’s name on a communication that might connote laziness or apathy. Organizations that are skillful in their management of stakeholder expectations and interactions with them can improve the value of the service from the view of those stakeholders. 

Much of this metaphor would suggest the performance of the actors in delivery. However, the scenery cannot be ignored. Just like an audience might be favorably impressed with superior stage setting, design, and props, the health care service delivery must be provided in a place that is clean, professional, and connotes the appropriate atmosphere. To that end, to be successful, service performance is about managing the expectations and meeting the needs of stakeholders. In one particular interdisciplinary collaborative, pharmacists worked with other healthcare professionals to manage the expectations of kidney transplants patients during medical and education services, as well as with self-monitoring activities, to better regulate their expectations and elevate their experiences throughout the process [87].

## 13. Monitoring and Sustaining Value-Added Pharmacist Services Through Implementation Science

Although there are many steps involved in getting a new service started, part of implementation activities also includes thinking about what needs to be achieved for sustainability (or not) [88]. Additionally, in the spirit of public health discussed previously, pharmacy organizations will advance the profession and best serve the community and its vulnerable populations when it develops sustainable services that treat and monitor patient progress for positive outcomes over the long haul. When approaching the value-added services concept, a useful paradigm to consider is implementation science, which is defined as the “scientific study of methods to promote the systematic uptake of research findings and other evidence-based practices into routine practice, and, hence, to improve the quality and effectiveness of health services” [89]. As pharmacy, continues to work towards advancing practice, implementation science offers a key to promote the systematic uptake of newer evidence-based practices and a frame on how to improve the quality and effectiveness of existing services, such as MTM [90].

Within implementation science, a useful approach for the implementation of value-added pharmacy services is the Consolidated Framework for Implementation Research (CFIR) model [91]. The model has designers of services consider the following to help ensure service longevity: evidence strength and quality advantage; adaptability; trialability; complexity; design quality and packaging; and cost. The CFIR and similar implementation science frameworks have demonstrated success in service design and monitoring. The aforementioned appointment-based model (ABM) can have an even more positive impact on the pharmacy and its patients when designed using a CFIR approach [44]. Use of an implementation model approach was associated with greater longevity for an pharmacist-driven asthma service in the community setting [92]. This type of framework might be especially valuable for other “non-routine” or “outside the box” types of pharmacy services, such as those associated with assisting breastfeeding mothers with their particular medication-taking needs [93] and mental health services [94]. In one study, success rates of MTM services (defined as completed and reimbursable claims) rose from 42.9% to 64% of attempts following the adoption of a CFIR framework [95]. Again, the adoption of CFIR and other implementation science frameworks approaches service design and implementation from many aspects, but principle among them is the mindset of ongoing evaluation and the need to ensure program quality.

The CFIR provides an excellent framework for executing services that promote positive outcomes for all stakeholders, thus potentiating its success well after its initial design. However, there are still additional considerations for ensuring long-term sustainability of a service even well after its implementation. Lennox et al. reviewed the implementation literature for successful program sustainability and produced a comprehensive model that involves patients/clients, infrastructure, staff training and support, maintaining organizational readiness, continuously raising the organization’s profile, and keep attuned to socioeconomic and political changes in force [96]. Shelton and Cooper concurred, emphasizing the dynamic nature of sustainability and thus the organization’s ability to adapt to change [97].

## 14. Receiving Payment for Value-Added Services

Ultimately, for a value-added service to be successful, payment must be received for it. This can be complex for value-added pharmacist services because traditionally payment for pharmacist dispensing services has been tied to a medication and pharmacists are not recognized as providers by most payers. A first step is to set a price for the service. In order to do this, one must determine all of the costs (e.g., personnel, equipment and supplies, space) related to delivery of the service. One paradigm that has been used is that a service should result in revenue that is two to three times a pharmacist’s salary [24]. Assuming that a pharmacist earns $60/hour, this may mean charging $2.00 to $3.00 per minute for a value-added pharmacy service [98]. It also important to remember that setting a price for a service does not mean that a payer (or patient) is willing to pay that price [24].

Next, various pricing methodologies should be reviewed to determine which one aligns best with the service. Fee-for-service pricing, which charges a set rate based on time or a specific intervention, is a common approach. However, it might not maximize revenue for the provider or might create disadvantages for promoting the service to patients. This strategy does not consider external factors, such as competitors’ prices and could also overestimate or underestimate its value to clients. Another pricing method is based on the resources used, or resource-based relative-value scale (RBRVS) used commonly in the U.S.^24^ There are available pharmacy-specific Current Procedural Technology (CPT) codes that might be accepted by Medicare carriers and other payers, including [99]:

99605: An initial encounter service performed face-to-face with a patient in a time increment of up to 15 min.

99606: For use for a subsequent or follow-up encounter with the same patient in a time increment of up to 15 min.

99607: An add-on code that may be used to bill for additional increments of 15 min of time to either of the above codes.

CPT code 99211 is another code which has been used by pharmacists providing patient care incident to a physician [99]. There are various codes for specific procedures such as for fasting lipid panel, finger stick, and others. In partnership with providers, pharmacists may also be able to use codes such as CPT code 99490 for chronic care management [99].

As mentioned previously, the use of implementation science and CFIR frameworks can assist not only in sustainability, but also with quality assurance and reimbursement for services. Increasingly, payers are looking for value; that is, payment for services that will save money for the insurer and its patients in the long term, when considering all medical costs. Alternative payment models reward performance, sustainment, and quality metrics. Therefore, it makes sense to identify and resolve medication-related problems “upstream” at the point-of-care to avoid clinical inertia, medication errors, and prevent more costly “downstream” care such as hospitalizations, readmissions, or emergency room visits [100]. This inertia, or momentum, often tempts pharmacy managers to focus on quantity (volume) and efficiency, which is important; however, reimbursement is often attained at higher levels and at higher rates when the goals of design and implementation focus on quality.

## 15. Conclusions

There are numerous opportunities for pharmacists to engage in the provision of valued-added services. Successful implementation of value-added services requires careful planning, evaluation and monitoring. The use of business and management principles as well as theory-based frameworks can facilitate a path to financial viability and sustainability of value-based pharmacist services.

## References

[1] Marcrom R.E., Horton R.M., Shepherd M.D. (1992). Creating value-added services to meet patient needs: Use these practical suggestions to help tailor your services to various market segments and expand your practice. J. Am. Pharm. Assoc..

[2] Westrick S.C. (2009). College/school of pharmacy affiliation and community pharmacies’ involvement in public health activities. Am. Assoc. Coll. Pharm. Annu. Meet..

[3] Queeno B.V. (2017). Evaluation of inpatient influenza and pneumococcal vaccination acceptance rates with pharmacist education. J. Pharm. Pract..

[4] Gonzalvo J.D., Lantaff W.M. (2018). CDE pharmacists in the United States. Diabetes Educ..

[5] Vargas J., Dang C., Subramaniam V. (2011). Public health approaches in ethnically diverse populations. Drug Top..

[6] Gerber B.S., Cano A.I., Caceres M.L., Smith D.E., Wilken L.A., Michaud J.B., Ruggiero L.A., Sharp L.K. (2010). A pharmacist and health promoter team to improve medication adherence among Latinos with diabetes. Ann. Pharmacother..

[7] Cochran G., Hruschak V., De Fosse B., Hohmeier K.C. (2016). Prescription opioid abuse: pharmacists’ perspective and response. Integr. Pharm. Res. Pract..

[8] Strand M.A., Eukel H., Burck S. (2019). Moving opioid misuse prevention upstream: A pilot study of community pharmacists screening for opioid risk. Res. Soc. Adm. Pharm..

[9] Hill L.G., Sanchez J.P., Laguado S.A., Lawson K.A. (2018). Operation naloxone: Overdose prevention service learning for student pharmacists. Curr. Pharm. Teach. Learn..

[10] Substance Abuse and Mental Health Services Administration (SAMHSA), Center for the Application of Prevention Technologies (2018). Preventing the Consequences of Opioid Overdose: Understanding Naloxone Access Laws. https://www.samhsa.gov/capt/sites/default/files/resources/naloxone-access-laws-tool.pdf.

[11] Fitzgerald T.J., Kang Y., Bridges C.B., Talbert T., Vagi S.J., Lamont B., Graitcer S.B. (2016). Integrating pharmacies into public health program planning for pandemic influenza vaccine response. Vaccine.

[12] Athern K.M., Linnebur S.A., Fabisiak G. (2016). Proper disposal of unused household medications: The role of the pharmacist. Consult. Pharm..

[13] Eerry L.A., Shinn B.W., Stanovich J. (2014). Quantification of an ongoing community-based medication take-back program. J. Am. Pharm. Assoc..

[14] Benedict A.W., Spence M.M., Sie J.L., Chin H.A., Ngo C.D., Salmingo J.F., Vidaurreta A.T., Rashid N. (2018). Evaluation of a Pharmacist-Managed Diabetes Program in a Primary Care Setting Within an Integrated Health Care System. J. Manag. Care Spec. Pharm..

[15] Choe H.M., Farris K.B., Stevenson J.G., Townsend K., Diez H.L., Remington T.L., Rockafellow S., Shimp L.A., Sy A., Wells T. (2012). Patient-centered medical home: Developing, expanding, and sustaining a role for pharmacists. Am. J. Health Syst. Pharm..

[16] Moczygemba L.R., Goode J.V., Gatewood S.B., Osborn R.D., Alexander A.J., Kennedy A.K., Stevens L.P., Matzke G.R. (2011). Integration of collaborative medication therapy management in a safety net patient-centered medical home. J. Am. Pharm. Assoc..

[17] Hirsch J.D., Steers N., Adler D.S., Kuo G.M., Morello C.M., Lang M., Singh R.F., Wood Y., Kaplan R.M., Mangione C.M. (2014). Primary-care based, pharmacist-physician collaborative medication-therapy management of hypertension: A randomized, pragmatic trial. Clin. Ther..

[18] Parente D.M., Morton J. (2018). Role of the pharmacist in antimicrobial stewardship. Med. Clin. N. Am..

[19] Baker S.N., Acquisto N.M., Ashley E.D., Fairbanks R.J., Beamish S.E., Haas C.E. (2011). Pharmacist-managed antimicrobial stewardship program for patients discharged from the emergency department. J. Pharm. Pract..

[20] Patel E., Pevnick J.M., Kennelty K.A. (2019). Pharmacists and medication reconciliation: A review of recent literature. Integr. Pharm. Res. Pract..

[21] Splawski J., Minger H. (2016). Value of the pharmacist in the medication reconciliation process. Pharm. Ther..

[22] Balch A.H., Constance J.E., Thorell E.A., Stockmann C., Korgenski E.K., Campbell S.C., Spigarelli M.G., Sherwin C.M. (2015). Pediatric vancomycin dosing: Trends over time and the impact of therapeutic drug monitoring. J. Clin. Pharmacol..

[23] Wong K.R., Nelson L.A., Elliott E.S., Liu Y., Sommi R.W., Winans E.A. (2016). Utilization of antipsychotic therapeutic drug monitoring at a state psychiatric hospital. Ment. Health Clin..

[24] Zgarrick D., Moczygemba L.R., Alston G.L., Desselle S.P. (2016). Pharmacy Management: Essentials for All Practice Settings.

[25] Feletto E., Wilson L.K., Roberts A.S., Benrimoj S.I. (2010). Building capacity to implement cognitive pharmaceutical services: Quantifying the needs of community pharmacies. Res. Soc. Adm. Pharm..

[26] Kennedy A.G., Biddle M.A. Practical Strategies for Pharmacist Integration with Primary Care: A Workbook. http://contentmanager.med.uvm.edu/docs/default-source/ahec-documents/practicalstrategiesforpharmacistintegrationwithprimarycare-workbook_000.pdf?sfvrsn=2.

[27] Cowart K., Olson K. (2019). Impact of pharmacist care provision in value-based care settings: How are we measuring value-added services?. J. Am. Pharm. Assoc..

[28] McAdam-Marx C., Dahal A., Jennings B., Singhal M., Gunning K. (2015). The effect of a diabetes collaborative care management program on clinical and economic outcomes in patients with type 2 diabetes. J. Manag. Care Spec. Pharm..

[29] American Pharmacists Association (APhA) (2016). Pharmacists’ Patient Care Services Digest. Building Momentum. Increasing Access..

[30] Smith M., Cannon-Breland M.L., Spiggle S. (2014). Consumer, physician and payer perspectives on primary care medication management services with a shared resource pharmacists network. Res. Soc. Adm. Pharm..

[31] Wu T., Williams C., Vranek K., Mattingly T.J. (2019). Using 340B discounts to provide a financially stable medication discharge service. Res. Soc. Adm. Pharm..

[32] Zrebiec J. (2014). A national study of the certified diabetes educator: Implications for future certification examinations. Diabetes Educ..

[33] Doucette W.R., McDonough R.P. (2002). Beyond the 4 P’s: Using relationship marketing to build value and demand for pharmacy services. J. Am. Pharm. Assoc..

[34] Feehan M., Walsh M., Godin J., Sundwall D., Munger M.A. (2017). Patient preferences for healthcare delivery through community pharmacy settings in the USA: A discrete choice study. J. Clin. Pharm. Ther..

[35] Painter J.T., Gressler L., Kathe N., Slabaugh L., Blumenschein K. (2018). Consumer willingness to pay for pharmacy services: An updated review of the literature. Res. Soc. Adm. Pharm..

[36] Nazar H., Nazar Z. (2019). Community pharmacy minor ailment services: Pharmacy stakeholder perspectives on the factors affecting sustainability. Res. Soc. Adm. Pharm..

[37] White L., Klinner C., Carter S. (2012). Consumer perspectives of the Australia Home Medicines Review Program: Benefits and barriers. Res. Soc. Adm. Pharm..

[38] Dabholkar P.A., Dunlap B. (2015). How to improve perceived service quality by increasing customer participation. Proceedings of the 1990 Academy of Marketing Science (AMS) Annual Conference. Developments in Marketing Science: Proceedings of the Academy of Marketing Science.

[39] Patwardhan P.D., Amin M.E., Chewning B.A. (2014). Intervention research to enhance community pharmacists’ cognitive services: A systematic review. Res. Soc. Adm. Pharm..

[40] Fleming M.L., Bapat S.S., Varisco T.J. (2019). Using the theory of planned behavior to investigate community pharmacists’ beliefs regarding engaging patients about prescription drug misuse. Res. Soc. Adm. Pharm..

[41] Holiday-Goodman M. (2012). Entrepreneurship, resource management, organizational culture and other “business” factors influencing pharmacy practice change. Res. Soc. Adm. Pharm..

[42] Treneman S., Willison M., Robinson B., Andrew M. (2019). A collaborative intervention for deprescribing: The role of stakeholder and patient engagement. Res. Soc. Adm. Pharm..

[43] Bitner M.J., Swartz T.A., Iacobucii D. (2000). The servicescape. Handbook of Services Marketing.

[44] Zeithaml V., Bitner M.J. (1996). Services Marketing.

[45] Goedken A.M., Butler C.M., McDonough R.P., Deninger M.J., Doucette W.R. (2018). Continuous medication monitoring (CoMM): A foundational model to support the clinical work of community pharmacists. Res. Soc. Adm. Pharm..

[46] (2008). Medication Therapy Management in Pharmacy Practice: Core Elements of an MTM Service Model, Version 2.0. American Pharmacists Association and National Association of Chain Drug Stores Foundation. https://www.pharmacist.com/sites/default/files/files/core_elements_of_an_mtm_practice.pdf.

[47] Patterson J., Holdford D. (2017). Understanding the dissemination of appointment-based synchronization models using the CFIR framework. Res. Soc. Adm. Pharm..

[48] Watson L.L., Bluml B.M. (2013). Pharmacy’s Appointment Based Model: Implementation Guide for Pharmacy Practices. APhA Foundation. https://www.aphafoundation.org/sites/default/files/ckeditor/files/ABMImplementationGuide-FINAL-20130923.pdf.

[49] Khazan E., Anastasia E., Hough A., Parra D. (2017). Pharmacist-managed ambulatory blood pressure monitoring service. Am. J. Health Syst. Pharm..

[50] Milfred-LaForest S.K., Gee J.A., Pugacz A.M., Pina I.L., Hoover D.M., Wenzell R.C., Felton A., Guttenberg E., Ortiz J. (2017). Heart failure transitions of care: A pharmacist-led post-discharge pilot experience. Prog. Cardiovasc. Dis..

[51] Halalau A., Shelden D., Keeney S., Hehar J. (2018). Pharm-MD; an open-label, randomized controlled, phase II study to evaluate the efficacy of a pharmacist-managed diabetes clinic in high-risk diabetes patients study protocol for a randomized controlled trial. Trials.

[52] Schultz J.L., Horner K.E., McDanel D.L., Miller M.L., Beranek R.L., Jacobsen R.B., Sly N.J., Miller A.C., Mascardo L.A. (2018). Comparing clinical outcomes of a pharmacist-managed diabetes clinic to usual physician-based care. J. Pharm. Pract..

[53] Pett R.G., Nye S. (2016). Evaluation of a pharmacist-managed asthma clinic in an Indian Health Service clinic. J. Am. Pharm. Assoc..

[54] Hale A., Merlo G., Nissen L., Coombes I., Graves N. (2018). Cost-effectiveness analysis of doctor-pharmacist collaborative prescribing for venous thromboembolism in high risk surgical patients. BMC Health Serv. Res..

[55] Lee J.C., Horner K.E., Krummel M.L., McDanel D.L. (2018). Clinical and financial outcomes evaluation of multimodal pharmacist warfarin management of a statewide urban and rural population. J. Pharm. Pract..

[56] Manzoor B.S., Cheng W.H., Lee J.C., Uppuluri E.M., Nutescu E.A. (2017). Quality of pharmacist-managed anticoagulation therapy in long-term ambulatory settings: A systematic review. Ann. Pharmacother..

[57] Durham M.J., Goad J.A., Neinstien L.S., Lou M.A. (2011). A comparison of pharmacist travel-health specialists’ versus primary care providers’ recommendations for travel-related medications, vaccination, and patient compliance in a college health setting. J. Travel Med..

[58] Hess K.M., Dai C.W., Garner B., Law A.V. (2010). Measuring outcomes of a pharmacist-run travel health clinic located within an independent community pharmacy. J. Am. Pharm. Assoc..

[59] Manchikanti Gomez A. (2017). Availability of pharmacist-prescribed contraception in California. J. Am. Med. Assoc..

[60] Puzantian T., Gasper J.J. (2018). Provision of naloxone without a prescription by California pharmacists 2 years after legislation implementation. J. Am. Med. Assoc..

[61] Smalls T.D., Broughton A.D., Hylick E.V., Woodard T.J. (2015). Providing medication therapy management for smoking cessation patients. J. Pharm. Pract..

[62] Salvig B.E., Gulum A.H., Walters S.A., Edwards L.B., Fourakre T.N., Marvin S.C., McKenzie M.S., Moseley M.V., Ansari I.J. (2016). Pharmacist screening for risk of osteoporosis in elderly veterans. Consult. Pharm..

[63] Smith M.C., Boldt A.S., Waston C.M., Zillich A.J. (2013). Effectiveness of a pharmacy care management program for veterans with dyslipidemia. Pharmacotherapy.

[64] Isho N.Y., Kachlic M.D., Marcelo J.C., Martin M.T. (2017). Pharmacist-initiated hepatitis C virus screening in a community pharmacy to increase awareness and link to care at the medical center. J. Am. Pharm. Assoc..

[65] Klepser D.G., Klepser M.E., Smith J.K., Dering-Anderson A.M., Nelson M., Pohren L.E. (2017). Utilization of influenza and streptococcal pharyngitis point-of-care testing in the community pharmacy practice setting. Res. Soc. Adm. Pharm..

[66] Grayson G., Shulman D., SwartZ T.A., Iacobucci D. (2000). Impression management in services marketing. Handbook of Services Marketing.

[67] Guhl D., Blankart K.E., Stargardt T. (2018). Service quality and perceived customer value in community pharmacies. Health Serv. Manag. Res..

[68] Desselle S.P. (2016). An in-depth examination of pharmacy technician worklife through an organizational behaviour framework. Res. Soc. Adm. Pharm..

[69] Ghorob A., Bodenheimer T. (2015). Building teams in primary care: A practical guide. Fam. Syst. Health.

[70] Brummel A., Lustig A., Westrich K., Evans M.A., Plank G.S., Penso J., Dubois R.W. (2014). Best practices: Improving patient outcomes and costs in an ACO through comprehensive medication therapy management. J. Manag. Care Pharm..

[71] MacKeigan L.D., Ljaz N., Bojarski E.A., Dolovich L. (2017). Implementation of a reimbursed medication review program: Corporate and pharmacy level strategies. Res. Soc. Adm. Pharm..

[72] Musselman K.T., Moczygemba L.R., Pierce A.L., Plum M.B., Brokaw D.K., Kelly D.L. (2017). Development and implementation of clinical pharmacist services within an integrated medical group. J. Pharm. Pract..

[73] American Pharmacists Association Pharmacist Scope of Services. https://www.pharmacist.com/sites/default/files/files/APhA%20-%20PAPCC%20Scope%20of%20Services.pdf.

[74] Collaborative Practice Agreements and Pharmacists’ Patient Care Services: A Resource for Pharmacists. National Center for Chronic Disease Prevention and Health Promotion. https://www.cdc.gov/dhdsp/pubs/docs/Translational_Tools_Pharmacists.pdf.

[75] Collaborative Practice Agreements: Resources and More NASPA. https://naspa.us/resource/cpa/.

[76] Collaborative Practice Agreements (CPA) and Pharmacists’ Patient Care Services APhA Foundation. https://www.aphafoundation.org/collaborative-practice-agreements.

[77] Farris K.B., Ashwood D., McIntosh J., DiPietro N.A., Maderas N.M., Landau S.C., Swegle J., Solemani O. (2010). Preventing unintended pregnancy. Pharmacists’ roles in practice and policy via partnerships. J. Am. Pharm. Assoc..

[78] Beal J.L., Plake K.S.I. (2019). Social and legislative shaping of access to to contraceptives and the pharmacist’s role: A literature review. Res. Soc. Adm. Pharm..

[79] Parsai S. (2016). Nutrition education improves patient care. J. Am. Pharm. Assoc..

[80] Harmon M., Pogge B., Boomershine V. (2014). Evaluation of a pharmacist-led, 6-month weight loss program for obese patients. J. Am. Pharm. Assoc..

[81] Matzke G.R., Czar M.J., Lee W.T., Moczygemba L.R., Harlow L.D. (2016). Improving health of at risk rural patients: A collaborative care model. Am. J. Health Syst. Pharm..

[82] McGee J.E., Love J.G., Festervand T.A. (2000). Competitive advantage and the independent retail pharmacy: The role of distinctive competencies. J. Pharm. Mark. Manag..

[83] Planas L.G. (2008). Intervention design, implementation, and evaluation. Am. J. Health Syst. Pharm..

[84] Delivering Medication Therapy Management Services American Pharmacists Association. https://www.pharmacist.com/education/advanced-training-programs/delivering-medication-therapy-management-services?is_sso_called=1.

[85] Jobin J., Irwin A.N., Pimentel J., Turner M.C. (2018). Accuracy of medication histories collected by pharmacy technicians during hospital admission. Res. Soc. Adm. Pharm..

[86] Grove S.J., Fisk R.P., John J., Swartz T.A., Iacobucci D. (2000). Services as theatre: Guidelines and implications. Handbook of Services Marketing and Management.

[87] Crawford C., Low J.K., Manias E., Williams A. (2017). Healthcare professionals can assist patients with managing post kidney transplant expectations. Res. Soc. Adm. Pharm..

[88] Crespo-Gonzalez C., Garcia-Cardenas V., Benrimoj S.I. (2017). The next phase in professional services research: From implementation to sustainability. Res. Soc. Adm. Pharm..

[89] Eccles M.P., Mittman B.S. (2006). Welcome to implementation science. Implement. Sci..

[90] Curran G.M., Shoemaker S.J. (2017). Advancing pharmacy practice through implementation science. Res. Soc. Adm. Pharm..

[91] Shoemaker S.J., Curran G.M., Swan H., Teeter B.S., Thomas J. (2017). Application of the Consolidated Framework for implementation research to community pharmacy: A framework for implementation research on pharmacy services. Res. Soc. Adm. Pharm..

[92] Fuller J.M., Saini B., Bosnic-Anticevich S., Cardenas V.G., Benrimoj S.I., Armour C. (2017). Testing evidence routine practice: Using an implementation framework to embed a clinically proven asthma service in Australian community pharmacy. Res. Soc. Adm. Pharm..

[93] Sim T.F., Hattingh H.L., Sherriff J., Tee L.B.G. (2017). Towards the implementation of breastfeeding-related health services in community pharmacies: Pharmacists’ perspectives. Res. Soc. Adm. Pharm..

[94] Hattingh H.L., Kelly F., Fowler J., Wheeler A.J. (2017). Implementation of a mental health medication management intervention in Australian community pharmacies: Facilitators and challenges. Res. Soc. Adm. Pharm..

[95] Stafford R., Thomas J., Payakachat N., Diemer T., Lang M., Kordsmeier B., Curran G. (2017). Using an array of implementation strategies to improve success rates of pharmacist-initiated medication therapy management services in community pharmacies. Res. Soc. Adm. Pharm..

[96] Lennox L., Maher L., Reed J. (2018). Navigating the sustainability landscape: A systematic review of sustainability approaches in healthcare. Implement. Sci..

[97] Shelton R.C., Cooper B.R., Stirman S.W. (2018). The Sustainability of Evidence-Based Interventions and Practices in Public Health and Health Care. Annu. Rev. Public Health.

[98] DaVanzo J., Dobson A., Koenig L., Book R. (2005). Medication Therapy Management Services: A Critical Review. https://www.acccp.com/docs/positions/commentarties/memts/pdf.

[99] Payment Methods in Outpatient Team-Based Clinical Pharmacy Practice, Part 1 (2018). ACCP Practice Advancement Issue Brief. https://www.accp.com/docs/positions/misc/IB1PaymentPart1-ACCPPracticeAdvancement.pdf.

[100] Smith M.A. (2017). Implementing primary care pharmacist services: Go upstream in the world of value-based payment models. Res. Soc. Adm. Pharm..

